# Type-specific EV-D68 real-time RT-PCR assay for the detection of all extant enterovirus D68 strains

**DOI:** 10.1128/jcm.01492-22

**Published:** 2025-08-20

**Authors:** Terry Fei Fan Ng, W. Allan Nix, Shannon L. Rogers, Brian Emery, Shur-Wern Chern, Kiantra Butler, M. Steven Oberste

**Affiliations:** 1 Division of Viral Diseases, Centers for Disease Control and Prevention 1242, Atlanta, Georgia, USA; Cepheid, Shanghai, China

**Keywords:** EV-D68, diagnostic assay, real-time PCR, rRT-PCR, Enterovirus D68

## Abstract

We developed a type-specific Enterovirus D68 (EV-D68) real-time RT-PCR (rRT-PCR) assay termed CDC2022, which targets sequences encoding conserved amino acid regions of all extant EV-D68 strains. We targeted three motifs conserved among all strains in the last 60 years. The assay achieved 100% (281/281) sensitivity and 100% (344/344) specificity when tested with a collection of 625 respiratory specimens, compared to the gold-standard EV semi-nested VP1 PCR and sequencing assay (snPCR/Seq). CDC2022 gave negative results with 289/289 non-target viruses, including 104 EV A-D isolates, 165 rhinovirus (RV) isolates or clinical specimens, and 14 other common respiratory viruses. The limit of detection (LOD) of the CDC2022 assay is 361 copies per reaction, as determined using serially diluted RNA transcripts. It can detect as few as 0.28 CCID_50_ per reaction with titrated isolates. An *in silico* “phylo-primer-mismatch" analysis was performed to visualize primer/probe mismatches and to compare CDC2022 with other EV-D68 rRT-PCR assays. It showed that CDC2022 has the fewest primer/probe mismatches among all assays analyzed and is suitable for all clades. As a type-specific assay targeting conserved amino acids, the CDC2022 assay allowed detection of newer strains from 2024. The CDC 2022 assay could provide a critical tool for molecular surveillance of EV-D68.

**IMPORTANCE:**

EV-D68 has caused recurring respiratory disease outbreaks in the United States since 2014. As recurrent outbreaks and continued virus evolution are expected for EV-D68, the CDC2022 rRT-PCR provides a robust test that detects known strains as well as potential emerging strains. This type-specific assay approach is critical for national EV-D68 surveillance and clinical diagnostics. An *in silico* “phylo-primer-mismatch" approach is invented to show EV-D68 assay robustness, but it has utility in new molecular tests for pathogen detection.

## INTRODUCTION

Enterovirus D68 (EV-D68; genus *Enterovirus*, family *Picornaviridae*) can cause severe respiratory illness, with clinical manifestations that include bronchiolitis, wheezing, and pneumonia, especially in children (1). EV-D68 has also been associated with acute flaccid myelitis (2–5). EV-D68 had been relatively rare from its discovery in 1962 until the mid-2000s (6), but more recently, it has been associated with clusters and outbreaks of severe respiratory disease worldwide (2, 7, 8). In 2014, EV-D68 caused a nationwide outbreak of severe respiratory tract illness in the United States, with over 1,100 laboratory-confirmed cases in 49 states and the District of Columbia. Successive outbreaks have occurred in the United States and many other countries in 2016 and 2018, suggesting a biannual pattern. A low level of circulation has been observed since 2019 (7), likely due to non-pharmaceutical interventions instituted to prevent the spread of SARS-CoV-2 (9).

Enteroviruses (EVs) other than EV-D68 do not usually cause respiratory diseases. At CDC, pan-EV real-time reverse-transcription polymerase chain reaction (rRT-PCR) (10) and EV VP1 semi-nested PCR and sequencing assays (snPCR/seq) (11) are the primary approaches for enterovirus diagnostics. While the gold-standard snPCR/seq method allows sensitive detection of all enteroviruses and many rhinoviruses, as well as the identification of the virus type, it is labor-intensive and difficult to scale to large numbers of specimens. To facilitate more rapid laboratory identification of EV-D68 cases during the 2014 outbreak investigation, an EV-D68-specific real-time PCR (rRT-PCR) assay was developed (https://www.fda.gov/media/120427/). The assay (termed CDC2015) targets the dominant strain that circulated in 2014, which is now defined as clade B1. Clade B1 continued to evolve, so by the time of the 2016 and 2018 outbreaks, the major circulating lineages were clades B2 and B3, but a few clade D cases have also been reported. Clade A has not been detected post-2014. As the sequences of the predominant circulating strains continue to evolve, the performance of CDC2015 has decreased (12, 13), likely due to sequence divergence compared to the original 2014 clade B1 outbreak strain. Other rRT-PCR assays, from Washington University (WU) (14) and from Niigata University (NU) (12) have been described for detection of the newer clades.

With the recurrence of EV-D68 circulation and continued evolution of the virus, a versatile EV-D68 rRT-PCR assay is needed to detect not only all known clades but also future, potentially divergent strains. Here, we describe a *pan*-EV-D68 rRT-PCR assay (called CDC2022) that detects all known EV-D68 clades. In addition to validation of the CDC2022 assay, we present *in silico* analyses of the CDC2015, CDC2022, WU, and NU assays to predict clade-specific characteristics of these commonly used EV-D68 rRT-PCR assays.

## MATERIALS AND METHODS

### Primer and probe design

**Fig 1:**
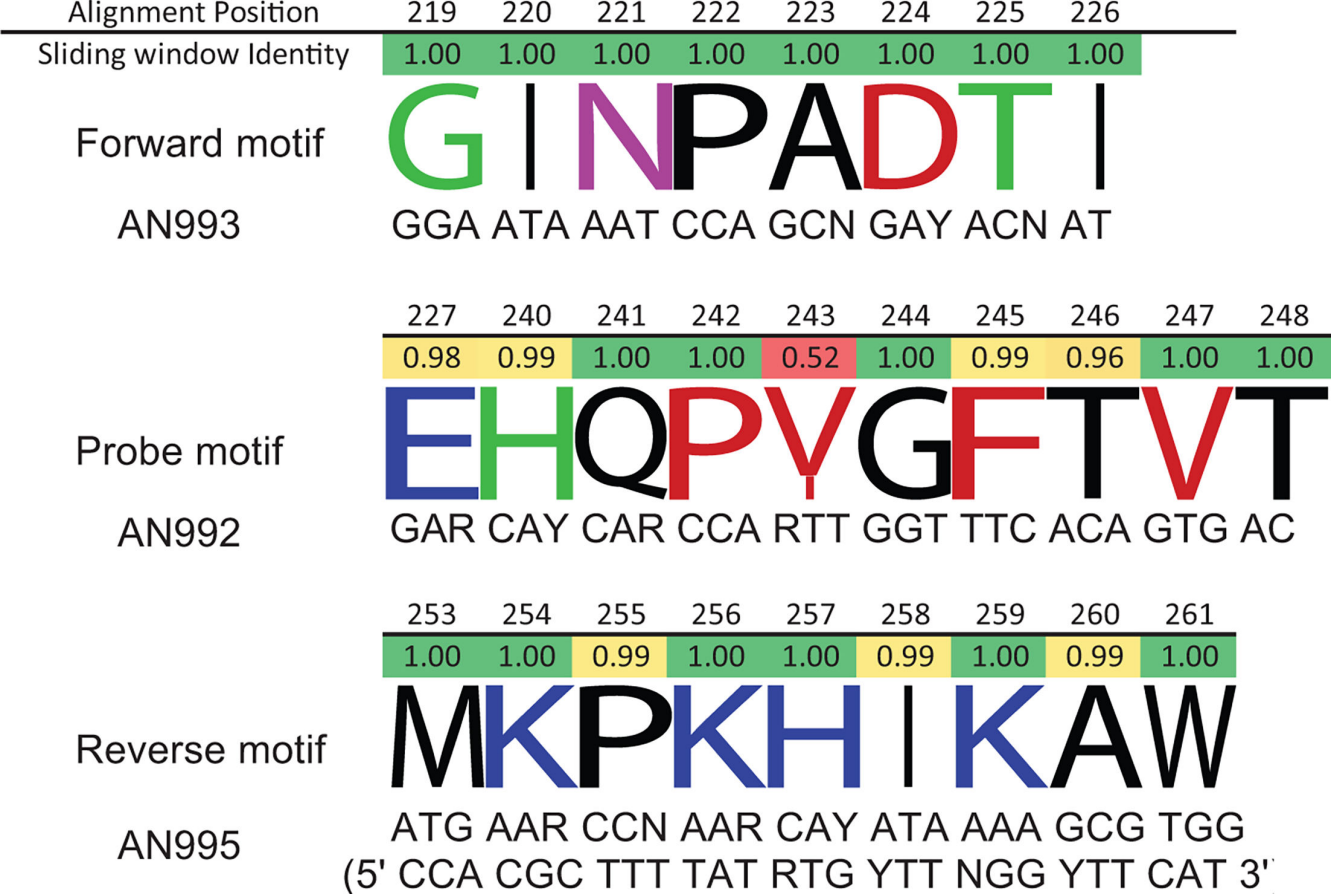
Conserved motifs targeted for CDC2022 pan-EV-D68 rRT-PCR.

To design the pan-EV-D68 primers (CDC2022), EV-D68 VP1 protein sequences were analyzed to identify regions of amino acid conservation (enterovirus VP1 contains immunodominant neutralization epitopes, and its sequence correlates with serotype [15]). Representative sequences from EV-D68 clades A, B, C, and D were obtained from GenBank, and sequences were manipulated using Geneious (versions including R9 and 2022.1.1). Specifically, only VP1-containing sequences with over 900 bp length were selected for further analysis. To reduce duplicative data, VP1 sequences with >99% nucleotide identity (NI) were excluded. VP1 nucleotide sequences were translated, and the amino acid sequences were aligned using MAFFT (16). Sequence logo and sliding window analysis with a window size of 30 amino acids was performed using Geneious to identify conserved amino acid motifs. As real-time PCR requires primers and a probe in close proximity to one another, selection of primer locations prioritized areas that are both conserved and close enough to one another to accommodate the constraints of a real-time PCR assay. Primer and probe sequences were manually designed on targeted conserved VP1 motifs using the nucleotide sequence alignment (Fig. 1), and primer properties including potential for primer-dimer formation were checked using Oligo Analyzer (IDTDNA).

### Real-time RT-PCR conditions

rRT-PCR for CDC2022 was performed using the qScript XLT One-Step RT-qPCR ToughMix (Quanta Biosciences; catalog #95132-500 or 95132-100). The reaction components and thermocycling conditions are provided in detail in Tables S1 and S2. Briefly, the 20 µL reaction mixture contained 10 µL of ToughMix enzyme reagent, 0.5 µL of 20 µM AN993 sense primer, 0.5 µL of 20 µM AN995 antisense primer, and 1 µL of 10 µM AN992 probe (0.5 µM final concentration of each primer and the probe), 3 µL of sterile water, and 5 µL of input RNA. Thermocycling and detection were performed using a 7500 Fast Real-Time PCR System (Applied Biosystems) according to the manufacturer’s instructions.

### RNA extraction

Virus isolates and respiratory specimens were extracted using the QIAgen Viral RNA Mini-Kit (QIAgen, Inc., Valencia, CA). Input sample volume was 140 µL, and RNA was eluted in 60 µL sterile nuclease-free water. Extracted nucleic acids were stored at −20°C until testing.

### Virus strains

EV-D68 isolates used in this study included Fermon prototype (GenBank accession number AY426531), US/MO/14-18947 (KM851225), US/MO/14-18949 (KM851227), US/2018-23087 (MK491180), and US/KY/14-18953 (KM851231) (17). EV-D68 isolates were grown in RD cells in 25 cm^2^ flasks and harvested at 60% to 70% cytopathic effect. The infected RD cells were frozen and thawed twice, and the supernatants were collected and clarified by centrifugation at 10,000 x *g* for 10 minutes, before aliquoting and freezing at −70°C. An aliquot of the virus was thawed and titrated on RD cell monolayers to determine the end point titer, expressed in units of 50% cell culture infectious dose (CCID_50_).

### Analytical sensitivity testing

To facilitate sensitivity evaluation, the Fermon prototype strain and representative EV-D68 isolates from clades B1, B2, B3, and D were spiked into minimum essential medium (MEM). Isolates were titrated before spiking. The RNA was then extracted and serially diluted to the range of 10^−5^ to 10^−9^. For each strain, each dilution was tested three times using the CDC2022 assay. A similar sensitivity evaluation was performed with the CDC2015 assay (Table S3) with a smaller isolate collection. The CDC 2022 assay sensitivity was further evaluated by spiking two representative isolates into EV-negative clinical nasopharyngeal/oropharyngeal swab matrix, with 20 technical replicates. Titrated clade B1 isolate US/MO/14-18949 and clade B2 isolate US/18-23087 were first serially diluted to the range of 10^−6^ to 10^−8^, and then dilutions were independently extracted 20 times and tested using the CDC2022 assay.

In addition to CCID_50_, an RNA transcript was generated *in vitro* to determine analytical sensitivity in terms of limit of detection (LOD) by RNA copy number. Briefly, EV-D68 amplicon containing the target VP1 and a T3-overhang was generated using RT-PCR. The purified amplicon was used as the template to produce transcript RNA using the MEGAscript T3 Transcription Kit (Invitrogen). The transcript RNA was purified using the MEGAclear kit (Invitrogen; including DNase steps) and its RNA concentration measured using NanoDrop (Thermo Fisher Scientific) for calculating the number of RNA copies per microliter. Using series of tenfold dilutions, transcripts representing 10,000 to 0.01 copies per reaction were tested by the CDC2020 rRT-PCR, with 10 replicates tested per dilution.

### Analytical specificity testing

The analytical specificity of CDC2022 was evaluated against viruses from *Enterovirus* species A–D and *Rhinovirus* species A–C and thirteen other common respiratory viruses (summarized in Table 4, enumerated in Tables S4 to S7). The EV A–D samples are isolates collected in the CDC enterovirus reference laboratory, including 22 EV-A, 56 EV-B, 17 EV-C, and 6 EV-D isolates. The closest sequence relatives to EV-D68 are other EV-D types, and currently, four other types are known. Three of the four non-EV-D68 EV-D types were tested (EV-D70, EV-D94, and EV-D111); EV-D120, isolated from gorillas and a chimpanzee, in Cameroon and the Democratic Republic of Congo, respectively, was not available for testing. To date, EV-D120 has not been detected in humans. In addition to the available “nearest neighbors” in EV-D, other common respiratory viruses were also tested. These included 95 rhinovirus (RV) isolates from species A and B (RV-A; RV-B), representing the classically defined serotypes, as well as adenovirus C1, coronaviruses (229E, OC43, and MERS-CoV), human metapneumovirus, influenza virus (A H1N1 and A H3N2 and B), parainfluenza viruses 1–3 and 4 a, respiratory syncytial virus, measles virus, and mumps virus. Rhinovirus species C (RV-C) (genus *Enterovirus*) have not been successfully grown in conventional cell cultures. RNA was extracted from 69 respiratory tract clinical specimens (nasopharyngeal/oropharyngeal swabs in viral transport medium) in which RV-C were identified by direct sequencing during 2014 and used to test CDC2022 assay specificity.

### Phylo-primer-mismatch analysis

Similar to the approach used for primer design, representative EV-D68 sequences were obtained from GenBank and were manipulated using Geneious (versions including R9 and 2022.1.1). VP1 sequences over 900 bp length were selected for analysis, and VP1 sequences with >99% nucleotide identity (NI) were excluded to reduce duplication. The 359 unique VP1 nucleotide sequences were aligned using MAFFT (16). For each assay, primer and probe sequences were mapped against the VP1 alignment to analyze the number of mismatches per strain. A neighbor-joining tree using the Jukes-Cantor substitution model was generated using Geneious. The mismatches were tabulated for visualization and overlaid with the phylogenetic tree.

### Testing of clinical specimens

A total of 625 respiratory specimens collected in 2012–2022 were tested in parallel using snPCR (gold standard) (11) and CDC2022 (Table S9). The partial VP1 sequences by snPCR were generated using the snPCR/Seq method (11) and typed using CDC PiType (https://pitype.cdc.gov/). In a sister published project, the NVSN network (18) has used this CDC2022 assay to screen 30,435 children with severe acute respiratory illness (ARI) from 2017 to 2022. For this report, additional samples from 2024 were further screened by CDC2022; they are confirmed with whole-genome next-generation sequencing instead of VP1-Sanger (unpublished result) and thus excluded in Table S9.

## RESULTS

### Primer and probe design

A comprehensive collection of EV-D68 VP1 clades A–D VP1 sequences were analyzed for conserved motifs suitable for a pan-EV-D68 rRT-PCR assay. Three neighboring conserved motifs, GINPADTI (sense primer), MKPKHIKAW (antisense primer), and EHQP(V/I)GFTVT (probe), were identified (Fig. 1). Each of these motifs is conserved for >99% of all available EV-D68 VP1 sequences. Sequence logo and sliding window analysis confirmed the same degree of sequence conservation (Fig. 1). Nucleotide degeneracy is incorporated in the 3′ half of the primers to account for all codons of the target protein motifs. The primer and probe sequences are described in Table 1.

**TABLE 1:** EV-D68 rRT-PCR CDC2022 and CDC2015 assay primers and probes^*a*^

Assay	Target	Description	Location (relative to Fermon)	Name	Sequence
CDC2022	All clades	Sense primer	3,052–3,074	AN993	5’ GGA ATA AAT CCA GCN GAY ACN AT 3’
		Antisense primer	3,171–3,145	AN995	5’ CCA CGC TTT TAT RTG YTT NGG YTT CAT 3’
		Probe	3,103–3,131	AN992	5’ FAM-GAR CAY CAR CCA RTT GGT TTC ACA GTG AC-BHQ1 3’
CDC2015	Clade B1	Sense primer	2,554–2,579	AN887	5’ CAA ACT CGC ACA GTG ATA AAY CAR CA 3’
		Antisense primer	2,825–2,797	AN893	5’ GTA TTA TTA CTA CTA CCA TTC ACN GCN AC 3’
		Probe	2,705–2,683	AN890	5’ FAM GTC CAT TTG AAA AAG TTC TTG TC BHQ1 3’

^*a*^ Positions were relative to EV-D68 prototype strain (Fermon; GenBank accession AY426531). Nucleotide degeneracy was noted according to IUPAC ambiguity codes.

### Phylo-primer-mismatch analysis to assess primer mismatches to predict assay performance

**Fig 2:**
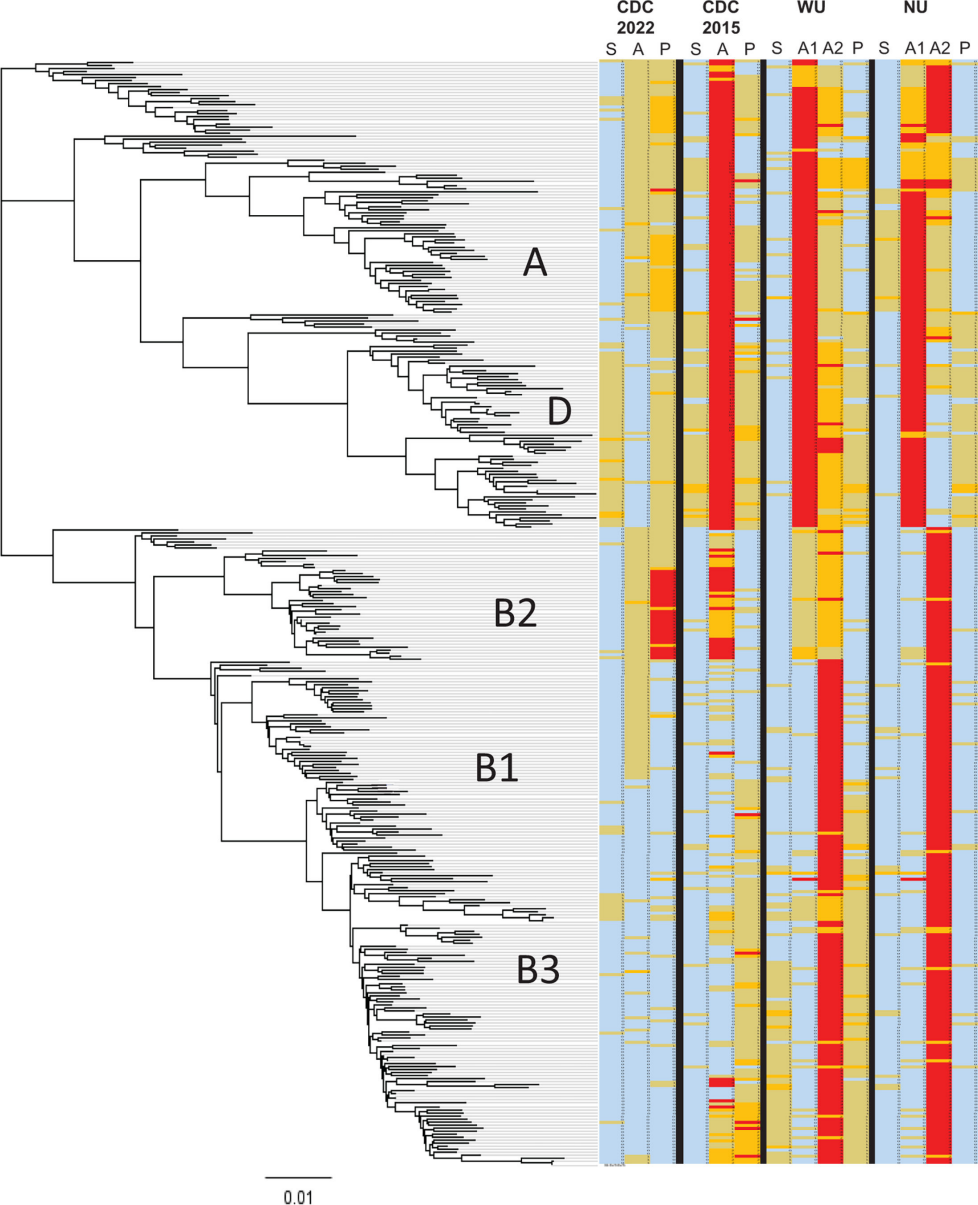
Phlyo-primer-mismatch graph for visualization of primer/probe mismatches against the EV-D68 phylogeny. Alignment of 359 representative deduplicated VP1 nucleotides was used to construct a neighbor-joining tree. Primer/probe mismatches to each sequence were tabulated and overlaid with the tree. Sense primer, antisense primer, and probe were evaluated in this order for each assay. WU and NU assays have two antisense primers. S, sense primer; A, antisense primer; A1, antisense-1 primer; A2, antisense-2 primer; P, probe. Blue, 0 mismatches; yellow, one mismatch; orange, two mismatches; red, three or more mismatches.

The primer and probe sequences for the CDC2015 and CDC2022 assays and other published EV-D68 assays (12, 14) were aligned with 359 unique, representative EV-D68 VP1 sequences (sequences with >99% nucleotide identities were deduplicated). We developed a “phylo-primer-mismatch graph,” depicting phylogenetic relationships among the reference sequences and degree of primer or probe mismatch, to visualize the number of sequence mismatches for each assay (Fig. 2). Sense primer, antisense primer, and probe were evaluated for each assay.

The CDC2022 sense and antisense primers and probe contain zero mismatches for most clade B strains, except for 27 clade B2 sequences that had 3–4 probe mismatches. They have ≤2 mismatches with clade A and D strains. In comparison, the CDC2015 assay has zero mismatches to most of the targeted clade B1 (range 0–3), but it contains 3–12 mismatches to sequences from clades A and D, and to historical strains from 1962 to the 2000s, including Fermon, as well as some clade B2 strains.

The phylo-primer-mismatch graph approach can visualize the primer-target relationships of existing assays to better understand their design. Both WU and NU assays contain two antisense primers (Fig. 2). The antisense-1 primer targets the clade B strains, while the antisense-2 primer targets clades A and D and the pre-2014 strains such as Fermon. Because the dual antisense primers allow either primer to bind to the target, the lowest mismatch of the two antisense primers should be considered when comparing primer/probe binding. For the WU assay, the antisense-1 primer targeting clade B contains zero mismatches to most clade B sequences (range 0–3). However, the antisense-2 targeting clades A and D contain two mismatches (range 0–3). The WU sense primer and probe contain a majority of 0 mismatches with clades B1 and B2 but had more mismatches (majority of 1–2 mismatches) with sequences in clade B3. The NU assay sense primer, antisense-1 primer, and probe have zero mismatches with most clade B sequences (range 0–3). The probe has one mismatch with most clade D sequences. The antisense-2 primer has more mismatches (1–4) with clade A and pre-2014 strains. Considering matched sequences with their primer/probe, the highest number of mismatches per virus sequence for CDC2022, CDC2014, WU, and NU are 4, 12, 7, and 6, respectively.

### Analytical sensitivity and specificity

The assay analytical sensitivity was evaluated by spiking the Fermon strain and representatives of EV-D68 clades B1, B2, B3, and D (Table 2) into MEM, followed by serial dilution and testing in the CDC2015 and CDC2022 assays. In MEM, the CDC2022 assay consistently detected as few as 10 CCID_50_/mL (0.28 CCID_50_ per reaction) of EV-D68 clades B1, B2, B3, D, and the Fermon strain (Table 2). The CDC2015 assay showed similar performance with clade B isolates (Table S3), but CDC2015 is at least 10 times less sensitive than CDC2022 for clade D and Fermon, likely due to the large number of mismatches identified in the phylo-primer-mismatch analysis.

**TABLE 2:** Analytic sensitivity of the CDC2022 EV-D68 rRT-PCR assays^*a*^

Assay	RNA	Fermon prototype	US/IL/14-18952	US/MO/14-18949	US/2018-23087	US/KY/14-18953
	Dilution	(historical)	(clade B1)	(clade B2)	(clade B3)	(clade D)
CDC2022						
	Undiluted stock CCID_50_	10^5.9^	10^8.0^	10^7.8^	10^7.1^	10^7.1^
	10-5	3/3 (8)	3/3 (1,000)	3/3 (630)	3/3 (125)	3/3 (125)
	10-6	**3/3** (**0.8**)	3/3 (100)	3/3 (63)	**3/3** (**12.5**)	3/3 (12.5)
	10-7	0/3 (0.08)	**3/3 (10**)	**3/3 (6**)	3/1 (1.25)	**3/3** (**1.25**)
	10-8	0/3 (0.008)	1/3 (1)	2/3 (0.6)	0/3 (0.125)	1/3 (0.125)
	10-9	0/3 (0.008)	0/3 (0.1)	0/3 (0.06)	0/3 (0.0125)	0/3 (0.0125)

^*a*^ Extracted isolate RNA was serially diluted and tested three times per strain. Detection rate was shown as positive / attempted. Virus titer (CCID_50_/mL) after dilution factor is shown in the parenthesis. The last dilution with 100% detection rate was shown in bold.

Similarly, by using 10-fold serially diluted RNA transcript, we determined the limit of detection (LOD) of the CDC2022 assay is 361 (copies per reaction) using probit analysis (Table 3C).

**TABLE 3:** Reproducibility and limit of detection (LOD) of EV-D68 rRT-PCR CDC2022 assay^*a*^

Dilution	CCID_50_/mL isolate	CCID_50_ per rRT-PCR	Call rate	Call rate %	Average Ct	Ct SD
A) EV-D68 virus isolate US/MO/14-18949						
10^−6^	63	0.7350	20/20	100%	32.8	0.69
10^−7^	6.3	0.0735	16/20	80%	36.4	2.1
10^−8^	0.63	0.0074	1/20	5%	NA	NA
B) EV-D68 virus isolate US/18-23087						
10^−6^	12.5	0.1458	20/20	100%	32.2	0.55
10^−7^	1.25	0.0146	14/20	70%	37.1	1.83
10^−8^	0.125	0.0015	4/20	20%	39	2.14

Copy per reaction	Call rate	Call rate %	Average Ct	Ct SD
C) EV-D68 RNA transcript				
10^4^	10/10	100%	29.6	0.3
10^3^	10/10	100%	33.3	0.2
10^2^	10/10	100%	37.8	0.4
10^1^	3/10	30%	40.5	0.9
10^0^	0/10	0%	NA	NA
10^−1^	0/10	0%	NA	NA
10^−2^	0/10	0%	NA	NA

^*a*^ A and B) limit of detection determined using isolate spiked clinical matrix (NP/OP). The pooled matrix spiked with isolates was serially diluted. Each dilution was extracted 20 times and tested. C) Limit of detection determined using EV-D68 RNA transcript; each dilution was tested 10 times. Using probit analysis, the LOD (361 copies per reaction) was determined. NA, not applicable.

The CDC2022 assay was also tested using virus-spiked enterovirus-negative respiratory specimen matrix (nasopharyngeal/oropharyngeal swab in the viral transport medium). The CDC2022 assay detected down to 63 CCID_50_/mL (0.735 CCID_50_ per reaction) for clade B1 strain 14-18949 (100% of 20 replicates) and 13 CCID_50_/mL (0.146 CCID_50_ per reaction) for clade B3 strain 18-23087 (100% of 20 replicates) (Table 3).

The analytical specificity of the pan-EV-D68 rRT-PCR (CDC2022) was evaluated by testing against a broad panel of viruses other than EV-D68, including *Enterovirus* species A–D (non-EV-D68 in EV-D) (Table S4), *Rhinovirus* species A–C (Tables S5 and S6), and 14 other common respiratory viruses (Table S7). All 289 non-EV-D68 viruses were negative using the CDC2022 assay, for an analytical specificity of 100% (summarized in Table 4).

**TABLE 4:** Analytic specificity of EV-D68 rRT-PCR CDC2022 assay with enteroviruses, rhinoviruses, and other respiratory viruses^*a*^

Cell culture isolates or respiratory clinical specimens	Summary	
	Negative/total	Specificity (%)
EV-D species	6/6	100
RV-A, -B, and -C species	165/165	100
EV-A, -B, and -C species	104/104	100
Other common respiratory viruses	14/14	100
Overall analytical specificity	289/289	100

^*a*^ The viruses tested are described in detail in Tables S4 to S7.

### CDC pan-EV-D68 rRT-PCR analytical sensitivity with clinical specimens

**Fig 3:**
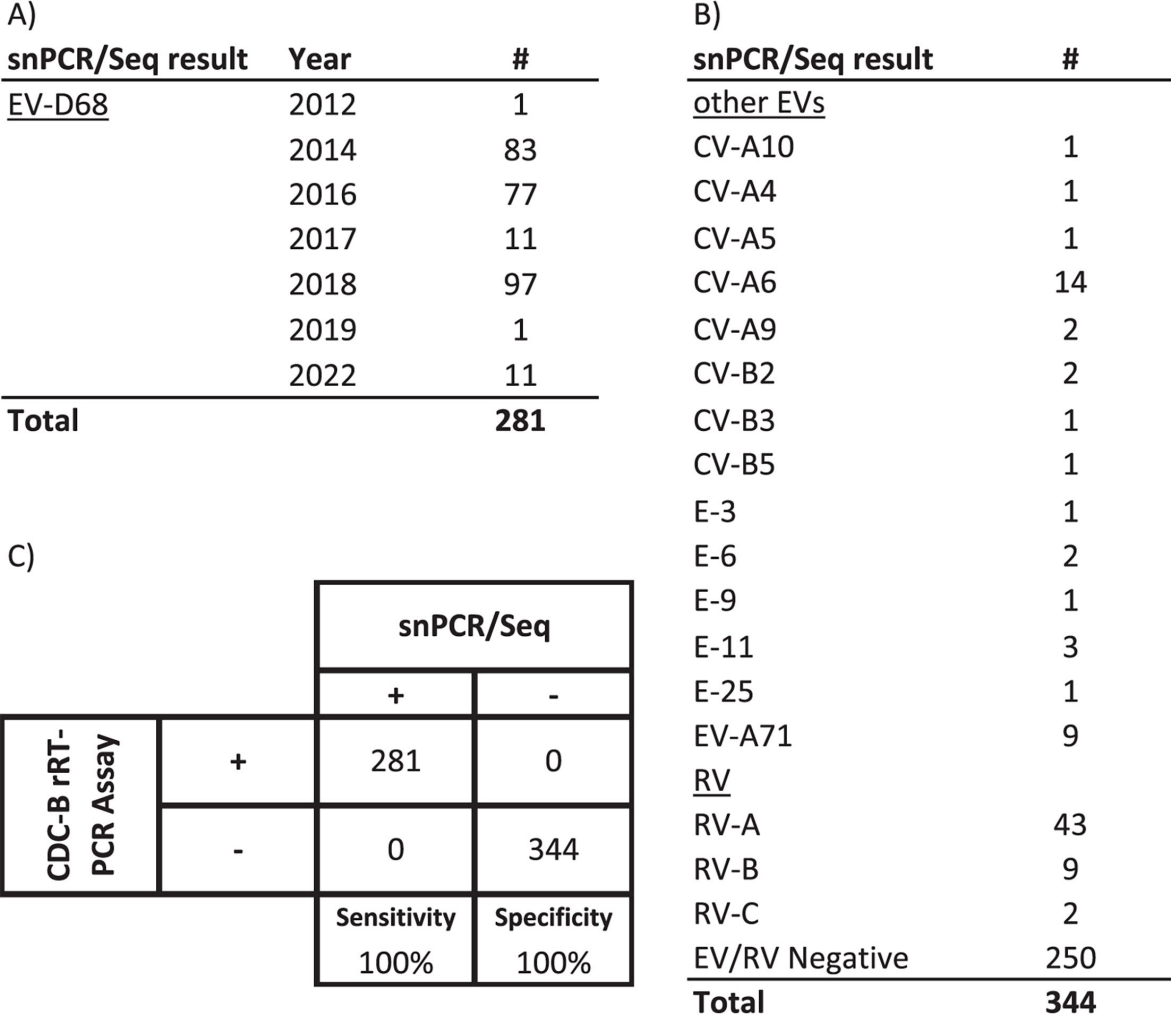
Clinical sensitivity of CDC-2022 rRT-PCR compared against the gold-standard snPCR/Seq. (**A**) Breakdown of the EV-D68-positive specimens tested by snPCR/Seq. (**B**) Breakdown of all other specimens tested and their snPCR/Seq result. (**C**) Confusion matrix of the rRT-PCR compared to snPCR/Seq showing the assay’s sensitivity and specificity in clinical specimens described in (**A and B**).

To evaluate the sensitivity of the CDC2022 assay in clinical specimens, we tested 625 respiratory specimens using the CDC2022 pan-EV-D68 rRT-PCR and the EV VP1 sequencing assay (snPCR/Seq). All 281 specimens that were EV-D68-positive by snPCR/Seq were detected by the CDC2022 assay, attaining 100% sensitivity (Fig. 3). The 344 snPCR/Seq EV-D68-negative samples (a combination of other enteroviruses and samples that were negative for all enteroviruses) were also negative with the CDC2022 assay (Table S9). Furthermore, the CDC2022 assay successfully detected EV-D68 in 20 clinical specimens from 2024 in which EV-D68 was confirmed by deep sequencing (unpublished result) as clade B3, the predominant clade that circulated during 2024. While beyond the scope of this paper’s data set, the published report from the NVSN network (18) has used the CDC2022 assay to detect 976 EV-D68 positives from screening 30,435 children with ARI from 2017 to 2022.

## DISCUSSION

Enterovirus infections are most frequently diagnosed by real-time RT-PCR targeting the highly conserved 5′-non-translated region of the viral genome (10). In most cases, identification of the enterovirus type is not necessary for clinical management of enterovirus disease and a result of “EV-positive” is a sufficient result, if only to help rule out other etiologies. Unfortunately, pan-EV assays tend to cross-react with rhinoviruses (and vice versa), making it difficult to differentiate between rhinovirus and enterovirus infection in respiratory disease (19). As a result, the available FDA-approved molecular diagnostic tests for testing of respiratory specimens usually report the result as “RV/EV positive.” However, enterovirus typing can be helpful in identifying disease clusters or outbreaks or when patients are being cohorted for infection control. In such cases, the method of choice is PCR amplification of a portion of the region that encodes the VP1 capsid protein, a region whose sequence correlates with the antigenic type, followed by sequencing (snPCR/Seq) (11). While the current VP1 snPCR/seq method is sensitive and can yield molecular epidemiologic data in addition to the virus type, these methods are relatively labor-intensive and do not scale well to large numbers of specimens. Such assays are also not widely available in state or local public health laboratories.

Type-specific real-time RT-PCR assays have been developed for several enteroviruses, including polioviruses (20), enterovirus A71 (21, 22), and coxsackievirus A16 (22). The major application of such assays is in disease surveillance to rapidly identify a specific virus of interest, such as in acute flaccid paralysis surveillance in support of global polio eradication (20) or during an outbreak in which a specific virus type is suspected or has been identified as the major etiologic agent (22). The major advantages of real-time PCR assays over sequencing are speed and scalability since an entire 96-well PCR plate can be quickly set up and run in only a few hours. This can be essential in an outbreak situation, such as the 2014 US EV-D68 outbreak, where the number of specimens can increase quickly, overwhelming the diagnostic laboratory. Since modern virus diagnostic laboratories are likely to already have real-time PCR instrumentation and trained staff for other virus diagnostic assays, the real-time PCR format makes it easy to rapidly deploy a new assay in diagnostic and public health laboratories.

The CDC 2015 assay was developed at the peak of the 2014 EV-D68 respiratory disease outbreak in the United States to facilitate more rapid differentiation of EV-D68 outbreak cases from the background of sporadic disease due to rhinoviruses and other enteroviruses (https://www.fda.gov/media/120427/). The CDC2015 assay sense and antisense primers had four-fold and 16-fold sequence degeneracy, respectively, to account for nucleotide variation in the outbreak strains and related historical sequences; the probe sequence is non-degenerate. We made a design decision for CDC2015 toward absolute sensitivity at the expense of breadth of reactivity, so the assay could be substituted for the sensitive gold standard assay, snPCR/seq at the time. As a result, the assay can detect EV-D68 sequences in clade B, but not the single outbreak virus that was in an outlier cluster (17), now known as Clade D. We considered this to be a reasonable compromise at the time, given that >99% of EV-D68 sequences from the 2014 outbreak were in clade B and were detected by the CDC2015 assay.

Since 2016, the major circulating EV-D68 strains have evolved to become clades B2 and B3, and several newer rRT-PCR assays were developed to detect the emerging strains (12, 14), yet some of the assays were shown to be less sensitive for certain clades (12). With the recurrence of EV-D68 outbreaks in recent years, we aimed to design a pan-EV-D68 rRT-PCR assay that detects all clades. By targeting conserved sites in the VP1 region, the assay should remain robust for detection of future EV-D68 sequence divergence. The sites encoding the GINPADTI amino acid motif (sense primer), the MKPKHIKAW motif (antisense primer), and the EHQP(V/I)GFTVT motif (probe) have been conserved among >99% of EV-D68 strains for the past 60 years, including the prototype strain, Fermon, and the most recent viruses in clade B3. The phylo-primer-mismatch analysis showed that the CDC2022 assay has 3–4 mismatches with several sequences from clade B2, but upon inspection, most of the mismatches are in the 5′ half of the probe and therefore have a lesser effect on probe binding. We believe the CDC2022 assay will provide a critical tool for molecular surveillance of EV-D68 as the virus continues to evolve, though ongoing strain surveillance will be required to ensure that is the case.

We developed a “phylo-primer-mismatch analysis” approach to visualize predicted primer and probe performance against a group of EV-D68 strains to assess the EV-D68 assays, but the approach could be applied to other pathogens as well. Although mismatches could be reported in the table format, the graphic overview provides useful insights into whether the mismatch(es) will affect primer/probe performance with specific virus clades. Through this analysis, we demonstrated the CDC2022 assay’s superiority over the CDC2015 assay in the breadth of the target range, in agreement with the assays’ performance in clinical testing. Primer mismatch analysis should not replace actual “wet-lab” comparison of assay performance, but it provides *in silico* evidence to guide assay design and to better understand observed assay performance. As currently implemented, the phylo-primer-mismatch analysis is largely a manual process, but a bioinformatic script that automates the analysis could be developed. Beyond enteroviruses, such a tool could help evaluate novel assay designs against an emergent strain or clade of a pathogen to ensure the primers and probe(s) are suitable for detecting all intended targets and account for sequence divergence. It can also be used to evaluate existing primers and probes against an ever-evolving virus.

This paper describes the evaluation of the CDC2022 rRT-PCR assay performance with a large specimen set for assessment of clinical sensitivity and specificity (*n* = 625) and analytical specificity (*n* = 289), providing validation of the assay’s robustness. *In silico* analysis showed CDC2022 has the fewest primer/probe mismatches among all assays evaluated. The NU assay also has very few primer/probe mismatches with the currently circulating B and D clades, which was supported by good assay performance (12), suggesting it is likely to perform well for currently circulating strains. However, unlike CDC2022, the NU and WU primers are not designed to account for target sequence evolution, and therefore they may fail to detect future EV-D68 strains. The CDC2022 assay targeted amino acid motifs that have been conserved for decades, making the assay resistant to future sequence divergence.

While rRT-PCR is an efficient way to diagnose EV-D68 in clinical cases, it does have limitations. The amplicon is too short for sequencing and therefore not ideal for molecular epidemiology or clade determination. Whole-genome amplification techniques (23), which may be more easily automatable than snPCR/seq, can be used as a reflex test for the rRT-PCR-positive specimens to generate genomes for molecular analysis. The whole-genome sequences, in turn, can help identify potential primer or probe mismatches and allow further improvement of the rRT-PCR assay.

## SUPPLEMENTAL MATERIAL

Supplemental tables jcm.01492-22-s0001.pdf Tables S1 to S9.

ASM does not own the copyrights to Supplemental Material that may be linked to, or accessed through, an article. The authors have granted ASM a non-exclusive, world-wide license to publish the Supplemental Material files. Please contact the corresponding author directly for reuse.
