# Supplementary material for: Type-specific EV-D68 real-time RT-PCR assay for the detection of all extant enterovirus D68 strains
Source: J Clin Microbiol. 2025 Aug 20;63(9):e01492-22. doi: 10.1128/jcm.01492-22 (PMC12421836; doi:10.1128/jcm.01492-22)
Supplement: Supplemental tables — Tables S1 to S9. [file jcm.01492-22-s0001.pdf]

Supplemental materials

Table S1. rRT-PCR reaction conditions for Applied Biosystems 7500 Fast.

Component	Single reaction
**2X ToughMix** enzyme reagent	10.0 µL
AN993 primer (20 µM)	0.5 µL
AN995 primer (20 µM)	0.5 µL
AN992 probe (10 µM)	1.0 µL
Sterile nuclease-free water	3.0 µL
Extracted nucleic acid	5.0 µL
Total volume	20.0 µL

Table S2. Thermocycling profile

Step	Cycles	Temperature	Time
Reverse transcription	1	50°C	30 min
Taq inhibitor inactivation	1	95°C	1 min
Polymerase chain reaction	45	95°C<br>52°C	15 sec<br>50 sec

Table S3. Analytic sensitivity of the EV-D68 rRT-PCR CDC2022 and CDC2015 assays.

**Assay**	**RNA Dilution**	**Fermon Prototype (historical)**	**US/IL/14-18952 (clade B1)**	**US/MO/14-18949 (clade B2)**	**US/2018-23087 (clade B3)**	**US/KY/14-18953 (clade D)**
**CDC2022**						
	**undiluted stock CCID50**	**$10^{5.9}$**	**$10^{8.0}$**	**$10^{7.8}$**	**$10^{7.1}$**	**$10^{7.1}$**
	**10-5**	3/3 (8)	3/3 (1000)	3/3 (630)	3/3 (125)	3/3 (125)
	**10-6**	**3/3 (0.8)**	3/3 (100)	3/3 (63)	**3/3 (12.5)**	3/3 (12.5)
	**10-7**	0/3 (0.08)	**3/3 (10)**	**3/3 (6)**	3/1 (1.25)	**3/3 (1.25)**
	**10-8**	0/3 (0.008)	1/3 (1)	2/3 (0.6)	0/3 (0.125)	1/3 (0.125)
	**10-9**	0/3 (0.008)	0/3 (0.1)	0/3 (0.06)	0/3 (0.0125)	0/3 (0.0125)
**CDC2015**						
	**10-5**	0/3 (8)	3/3 (1000)	3/3 (630)	not tested	**3/3 (125)**
	**10-6**	0/3 (0.8)	3/3 (100)	3/3 (63)	not tested	0/3 (12.5)
	**10-7**	0/3 (0.08)	**3/3 (10)**	3/3 (6)	not tested	0/3 (1.25)
	**10-8**	0/3 (0.008)	2/3 (1)	**3/3 (0.6)**	not tested	0/3 (0.125)
	**10-9**	0/3 (0.008)	0/3 (0.1)	1/3 (0.06)	not tested	0/3 (0.0125)

CDC2022 assay specificity

Enterovirus species A, B and C. Most EV types are not thought of as frank respiratory pathogens; however, most EV species A, B, and C do replicate to some extent in the nasopharynx and some types other than EV-D68 can cause acute respiratory disease, e.g., CV-A21 and EV-A71. Because of this, we investigated all the human (and non-human primate) EV species A-C for cross reactivity (**Table S4**). RNA was extracted from cell culture supernatants and tested undiluted. The Pan-EV-D68 rRT-PCR did not amplify any viruses from EV-A, EV-B, or EV-C.

Enterovirus Species D. To evaluate the analytical specificity of the Pan-EV-D68 rRT-PCR, we tested a panel of enterovirus species D (EV-D; five known types including EV-D68) cell culture isolates. Respiratory clinical specimens positive for EV-D70, EV-D94, EV-D111 were not available for testing, therefore RNA from these viruses was extracted from frozen undiluted cell culture supernatants and tested. The Pan-EV-D68 rRT-PCR did not amplify other *Enterovirus D* viruses (**Table S4**).

Table S4 Pan-EV-D68 rRT-PCR analytical specificity confirmed using undiluted RNA extracted from enterovirus cell culture supernatants

Species	Enterovirus Types	Pan-EV-D68 rRT-PCR Result
**EV-A**	Coxsackievirus A2 (CV-A2), CV-A3, CV-A4, CV-A5, CV-A6, CV-A7, CV-A8, CV-A10, CV-A12, CV-A14, CV-A16, Enterovirus A71 (EV-A71), EV-A76, EV-A89, EV-A90, EV-A91, EV-A92, EV-A114, EV-A122, EV-A123, EV-A124, EV-A125	All Negative (22/22)
**EV-B**	Coxsackievirus B1 (CV-B1), CV-B2, CV-B3, CV-B4, CV-B5, CV-B6, CV-A9, Echovirus 1 (E-1), E-2, E-3, E-4, E-5, E-6, E-7, E-9, E-11, E-12, E-13, E-14, E-15, E-16, E-17, E-18, E-19, E-20, E-21, E-24, E-25, E-26, E-27, E-29, E-30, E-31, E-32, E-33, enterovirus B69 (EV-B69), EV-B73, EV-B74, EV-B75, EV-B77, EV-B78, EV-B79, EV-B80, EV-B81, EV-B83, EV-B84, EV-B85, EV-B86, EV-B87, EV-B88, EV-B97, EV-B100, EV-B101, EV-B111, EV-B112, EV-B114	All Negative (56/56)
**EV-C**	Poliovirus (PV) 1, PV-2, PV-3, (and Sabin PV 1, 2, 3), coxsackievirus A1 (CV-A1), CV-A11, CV-A13, CV-A17, CV-A19, CV-A20, CV-A21, CV-A22, CV-A24, EV-C96, EV-C99, EV-C102, EV-C105, EV-C113	All Negative (21/21)
**EV-D (except EV-D68)**	EV-D70 (prototype J670/1971), EV-D94 (Nigeria, 2010 [two different isolates]), EV-D111 (Angola, 2012 [two different isolates])	All Negative (5/5)

[a] Three EV-A types, EV-A119, A120, and A121, are not available in CDC's collection or in public biorepositories. *In silico* examination of the missing EV-A nucleic acid sequences indicated that these viruses would not be detected by the pan-EV-D68 rRT-PCR assay.

[b] Seven EV-B types, EV-B82, B93, B98, B106, B107, B110, and B113, are not available in CDC's collection or in public biorepositories. *In silico* examination of the missing EV-B nucleic acid sequences indicated that these viruses would not be detected by the pan-EV-D68 rRT-PCR assay.

[c] Six EV-C types, EV-C95, C104, C109, C116, C117, and C118, are not available in CDC's collection or in public biorepositories. *In silico* examination of the missing EV-C nucleic acid sequences indicated that these viruses would not be detected by the pan-EV-D68 rRT-PCR assay.

[d] EV-D120, isolated from gorillas and a chimpanzee, in Cameroon and the Democratic Republic of Congo, respectively, was not available for testing. *In silico* examination of the EV-D120 nucleic acid sequences indicated that this virus would not be detected by the pan-EV-D68 rRT-PCR assay. To date, there are no reports of EV-D120 detection in humans.

Rhinovirus Species A and B. Rhinoviruses (RV) (genus *Enterovirus*) circulate widely during EV season and are commonly present in respiratory specimens. Therefore, we tested available RVs, using undiluted RNA extracted from frozen cell culture supernatants. A total of 96 culture-propagated RV, including 71 species A (RV-A) and 25 species B (RV-B) were tested (**Table S5**). The Pan-EV-D68 rRT-PCR did not amplify any viruses from RV-A or RV-B.

Table S5: Pan-EV-D68 rRT-PCR analytical specificity confirmed using undiluted RNA extracted from rhinovirus (RV) cell culture supernatants

Species	Rhinovirus Types	Pan-EV-D68 rRT-PCR Result
**RV-A**	RV-A1, A2, A7, A8, A9, A10, A11, A12, A13, A15, A16, A18, A19, A20, A21, A22, A23, A24, A25, A28, A29, A30, A31, A32, A33, A34, A36, A38, A39, A40, A41, A43, A45, A46, A47, A49, A50, A51, A53, A54, A55, A56, A57, A58, A59, A60, A61, A62, A63, A64, A65, A66, A67, A68, A71, A73, A74, A75, A76, A77, A78, A80, A81, A82, A85, A88, A89, A90, A94, A96, A100	All Negative (71/71)
**RV-B**	RV-B3, B4, B5, B6, B14, B17, B26, B27, B35, B37, B42, B48, B52, B69, B70, B72, B79, B83, B84, B86, B91, B92, B93, B97, B99	All Negative (25/25)

[a] Nine RV-A types, RV-A101, A102, A103, A104, A105, A106, A107, A108, and A109, are not available in CDC's collection or in public biorepositories. *In silico* examination of the missing RV-A nucleic acid sequences indicated that these viruses would not be detected by the pan-EV-D68 rRT-PCR assay.

[b] Seven RV-B types, RV-B100, B101, B102, B103, B104, B105, and B106, are not available in CDC's collection or in public biorepositories. *In silico* examination of the missing RV-B nucleic acid sequences indicated that these viruses would not be detected by the pan-EV-D68 rRT-PCR assay.

Rhinovirus Species C. Rhinovirus species C (RV-C) (genus *Enterovirus*) have not been successfully grown in conventional cell cultures. Sixty-nine RV-C, identified by direct sequencing of clinical specimens during 2014, were used to test specificity. Extracted RNA from respiratory tract clinical specimens (nasopharyngeal/oropharyngeal swabs in viral transport medium), containing RV-C types, was used to test the pan-EV-D68 rRT-PCR assay specificity. RV-C types in the NP/OP specimens were determined by Sanger sequencing of the VP1 region or by sequencing the RV VP4/VP2 region. The Pan-EV-D68 rRT-PCR did not amplify any of the RV-C viruses tested (**Table S6**).

Table S6: Pan-EV-D68 rRT-PCR analytical specificity confirmed using RV-C clinical specimen RNA

Enterovirus Species	Rhinovirus Types	Pan-EV-D68 rRT-PCR Result
**RV-C**	RV-C2, C3, C4, C5, C6, C7, C9, C10, C11, C12, C14, C16, C18, C19, C21, C22, C23, C24, C25, C26, C27, C28, C29, C30, C31, C32, C33, C34, C35, C37, C38, C39, C40, C41, C42, C43, C44, C45	All Negative (69/69)

[a] Eighteen RV-C types, RV-C1, C8, C13, C15, C17, C20, C36, C46, C47, C48, C49, C50, C51, C52, C53, C54, C55 and C56, are not available in CDC's collection or in public biorepositories. -*In silico* examination of the missing RV-C nucleic acid sequences indicated that these viruses would not be detected by the pan-EV-D68 rRT-PCR assay.

Other common respiratory viruses. Extracted RNA from fourteen common respiratory viruses was tested using the pan-EV-D68 rRT-PCR. RNA was extracted from viral cell culture supernatants and tested undiluted. The Pan-EV-D68 rRT-PCR did not amplify any of the tested viruses (**Table S7**).

Table S7: Pan-EV-D68 rRT-PCR cross-reactivity with common respiratory viruses

Virus[a]	Pan-EV-D68 rRT-PCR Result
**Adenovirus C1 (AdV-71)**	Negative
**CoV 229E**	Negative
**CoV OC43**	Negative
**CoV MERS**	Negative
**SARS-CoV-2**	Negative
**HMPV (CAN99-81)**	Negative
**Influenza A H1N1**	Negative
**Influenza A H3N2**	Negative
**Influenza B (B/Vic)**	Negative
**PIV1 (C35)**	Negative
**PIV2 (Greer)**	Negative
**PIV3 (C-43)**	Negative
**PIV4a (CH 19503)**	Negative
**RSV-A (Long)**	Negative

[a] CoV, coronavirus; HMPV, human metapneumovirus; PIV, parainfluenza virus; RSV, respiratory syncytial virus.

Other organisms commonly detected in respiratory tract specimens. Additional evaluation of the analytical specificity of the pan-EV-D68 rRT-PCR was performed through *in silico* analysis of the CDC2015 primer and probe sequences against common causes of respiratory illness and other clinically relevant organisms. BLASTn analysis queries of the VP1.2020 primers and probe were performed against the GenBank public domain nucleotide sequences and showed no significant combined homologies (primer targets and probe target) with other conditions that would predict potential false positive rRT-PCR results.

Table S8: CDC2015 primer and probe set *in silico* specificity analysis

Disease/condition	Taxid	Agent
**Human parainfluenza**	11226	Parainfluenza virus 4b
**Human parechovirus**	12063	Parechovirus A1
**Common cold, bronchiolitis, pneumonia**	208895	Respiratory syncytial virus B
**Sinusitis**	1280	*Staphylococcus aureus*
**Pertussis (whooping cough)**	520	*Bordetella pertussis*
**Mycoplasma pneumonia**	2104	*Mycoplasma pneumoniae*
**Pharyngitis, bronchitis, atypical pneumonia**	83558	*Chlamydia pneumoniae* (or *Chlamydophila pneumonia*)
**Bacteremia, pneumonia, epiglottitis and acute bacterial meningitis**	727	*Haemophilus influenza*
**Tuberculosis**	1773	*Mycobacterium tuberculosis*
**Lyme disease**	139	*Borrelia burgdorferi*
**Respiratory infection**	550	*Enterobacter cloacae*
**Septicaemia**	1354	*Enterococcus hirae*
**Foodborne illness**	1502	*Clostridium perfringens*
**Abdominal infection (rare)**	46503	*Parabacteroides merdae*
**Human gastrointestinal bacterium**	1680	*Bifidobacterium adolescentis*
**Human gastrointestinal bacterium**	216816	*Bifidobacterium longum*
**Human gastrointestinal bacterium**	40518	*Ruminococcus bromii*

Table S9. Line-by-line description of respiratory clinical samples tested by both snPCR/Seq and CDC2022 rRT-PCR.

Aspirate, ASP; bronchial wash, BW; nasal aspirate, NA; nasal swab, NAS; nasopharyngeal swab, NP; nasopharyngeal aspirate, NPA; nasal secretions, NS; nasal wash, NW; sputum, SP; trachial aspirate, TA; tracheal aspirate, TRA; throat swab, TS; throat wash, TW.

Number	Specimen type	VP1 RT-snPCR/Sequencing Result	Pan-EV-D68 CDC2022 Result (Ct or positivity)	Year
1	NP	E-11	Negative	2015
2	NP	CV-A6	Negative	2015
3	NP	RV-80	Negative	2015
4	NP	CV-A6	Negative	2015
5	NP	CV-A4	Negative	2015
6	NP	E-11	Negative	2015
7	NP	CV-A6	Negative	2015
8	NP	E-3	Negative	2015
9	NP	E-25	Negative	2016
10	NP	Negative	Negative	2016
11	NP	RV-A57	Negative	2016
12	NP	Negative	Negative	2016
13	NP	CV-A6	Negative	2016
14	NP	RV-A51	Negative	2016
15	NP	RV-A21	Negative	2016
16	NP	CV-A6	Negative	2016
17	NP	Negative	Negative	2016
18	NP	Negative	Negative	2016
19	NP	CV-A9	Negative	2016
20	NP	CV-A9	Negative	2016
21	NPA	E-6	Negative	2016
22	NP	Negative	Negative	2016
23	NP	CV-A6	Negative	2016
24	NP	Negative	Negative	2016
25	NP	RV-A49	Negative	2016
26	NP	Negative	Negative	2016
27	NP	Negative	Negative	2016
28	NP	CV-A6	Negative	2016
29	NP	RV-A20	Negative	2016
30	NP	RV-A12	Negative	2016
31	NP	E-6	Negative	2016
32	NP	Negative	Negative	2016
33	NP	Negative	Negative	2017
34	NP	Negative	Negative	2017
35	NP	RV-A49	Negative	2017
36	NP	CV-B3	Negative	2017
37	NP	Negative	Negative	2017
38	NP	Negative	Negative	2017
39	NP	Negative	Negative	2017
40	NP	Negative	Negative	2017
41	NP	RV-A101	Negative	2017
42	NP	RV-A56	Negative	2017
43	NP	RV-A55	Negative	2017
44	NP	CV-A6	Negative	2017
45	NP	CV-B2	Negative	2017
46	NP	RV-A1B	Negative	2017
47	NP	Negative	Negative	2017
48	NP	CV-B5	Negative	2017

49	NP	RV-A24	Negative	2017
50	NP	RV-A45	Negative	2018
51	NP	EV-D68	19.9	2014
52	NP	EV-D68	26.1	2014
53	NP	Negative	Negative	2014
54	NP	EV-D68	29.8	2014
55	NP	EV-D68	17.6	2014
56	NP	Negative	Negative	2014
57	NP	Negative	Negative	2014
58	NP	EV-D68	18.7	2014
59	NP	Negative	Negative	2014
60	NP	Negative	Negative	2014
61	NP	EV-D68	27.6	2014
62	NP	EV-D68	29.5	2014
63	NP	EV-D68	29.2	2014
64	NP	Negative	Negative	2014
65	NP	EV-D68	26.7	2014
66	NP	EV-D68	16.8	2014
67	NP	EV-D68	23.2	2014
68	NP	EV-D68	29.9	2014
69	NP	EV-D68	21.8	2014
70	NP	RV-B27	Negative	2014
71	NP	EV-D68	20.7	2014
72	NP	EV-D68	27.9	2014
73	NP	EV-D68	19.6	2014
74	NP	EV-D68	32.3	2014
75	NP	Negative	Negative	2014
76	NP	EV-D68	31.4	2014
77	NP	Negative	Negative	2014
78	NP	EV-D68	22.5	2014
79	NP	EV-D68	23	2014
80	NP	Negative	Negative	2014
81	NP	EV-D68	27.8	2014
82	NP	EV-D68	19.1	2014
83	NP	EV-D68	23.5	2014
84	NP	RV-A101	Negative	2014
85	NP	RV-A101	Negative	2014
86	NP	CV-A6	Negative	2014
87	NP	Negative	Negative	2014
88	NP	EV-D68	15.9	2014
89	NP	CV-A6	Negative	2014
90	NP	RV-77	Negative	2014
91	NP	EV-A71	Negative	2014
92	NP	EV-D68	20.2	2014
93	NP	EV-D68	15.6	2014
94	NP	EV-D68	24.9	2014
95	NPA	EV-D68	20.1	2014
96	NP	EV-D68	20.2	2014
97	NP	EV-D68	20.4	2014
98	NP	EV-D68	30.6	2014
99	NP	EV-D68	26.2	2014
100	NP	RV-59	Negative	2014
101	NP	RV-A94	Negative	2014
102	NP	EV-D68	29.1	2014
103	NP	EV-D68	31.9	2014

104	NP	EV-D68	28.9	2014
105	NP	EV-D68	26.4	2014
106	NP	EV-D68	21.8	2014
107	NP	EV-D68	28.3	2014
108	NP	EV-D68	25	2014
109	NP	EV-D68	26.8	2014
110	NP	EV-D68	35.6	2014
111	NP	RV-A34	Negative	2014
112	NP	RV-A63	Negative	2014
113	NP	EV-D68	29.9	2014
114	NP	RV-A49	Negative	2014
115	OP	E-9	Negative	2014
116	NP	EV-D68	21	2014
117	NP	EV-A71	Negative	2014
118	NP	EV-D68	25.1	2014
119	NP	RV-A24	Negative	2014
120	NP	EV-D68	18.2	2014
121	NP	EV-D68	20.9	2014
122	NP	EV-D68	18.4	2014
123	NP	EV-D68	15.6	2014
124	NP	EV-D68	28.9	2014
125	NW	EV-D68	23.8	2014
126	NP	RV-C	Negative	2014
127	NP	RV-C	Negative	2014
128	NP	EV-D68	28.6	2014
129	NP	RV-B6	Negative	2014
130	NP	RV-B6	Negative	2014
131	NW	RV-A101	Negative	2014
132	NP	EV-D68	20.5	2014
133	NP	EV-D68	24.3	2014
134	NP	EV-D68	27.5	2014
135	NP	EV-D68	22	2014
136	NP	EV-D68	23.9	2014
137	NP	RV-A73	Negative	2014
138	NP	Negative	Negative	2014
139	NP	EV-D68	29.2	2014
140	NP	RV-B48	Negative	2014
141	NP	CV-A10	Negative	2014
142	NP	EV-D68	27.3	2014
143	NP	EV-D68	21.4	2014
144	NP	EV-D68	28.9	2014
145	NP	EV-D68	26.2	2014
146	NP	EV-D68	26.6	2014
147	NP	EV-D68	29.8	2014
148	NP	EV-D68	17.4	2014
149	NP	EV-D68	33.5	2014
150	NP	EV-D68	32.5	2014
151	NP	EV-D68	31.1	2014
152	NP	EV-D68	25.3	2014
153	NP	EV-D68	24.8	2014
154	NP	EV-D68	19.9	2014
155	NP	EV-D68	17.2	2014
156	NP	Negative	Negative	2014
157	NP	Negative	Negative	2014
158	NP	EV-D68	21.5	2014

159	NP	Negative	Negative	2014
160	NP	EV-D68	15.7	2014
161	NP	Negative	Negative	2014
162	NP	EV-D68	31.3	2014
163	NP	EV-D68	23	2014
164	NP	EV-D68	20.3	2014
165	NP	EV-D68	26.3	2014
166	NP	EV-D68	30.5	2014
167	NP	EV-D68	33.3	2014
168	NP	EV-D68	34	2014
169	NP	EV-D68	21.6	2014
170	NP	EV-D68	24.5	2014
171	NP	RV-B37	31.5	2014
172	NP	Negative	Negative	2014
173	NP	Negative	Negative	2014
174	NP	Negative	Negative	2014
175	NP	EV-D68	31.1	2014
176	NP	EV-D68	29.2	2016
177	NP	EV-D68	30.3	2016
178	NP	EV-D68	33.9	2016
179	TRA	EV-D68	32.7	2016
180	NP	EV-D68	32.6	2016
181	NAS	EV-D68	38.9	2016
182	NP	EV-D68	32	2016
183	NP	EV-D68	30	2016
184	NP	EV-D68	35.5	2016
185	NP	EV-D68	29.4	2016
186	ASP	EV-D68	26.6	2016
187	NP	EV-D68	27	2016
188	NP	EV-D68	21.6	2016
189	NP	EV-D68	28.4	2016
190	NP	EV-D68	25.4	2016
191	NP	EV-D68	22	2016
192	BW	EV-D68	15	2016
193	NPA	EV-D68	27.9	2016
194	NPA	EV-D68	20.1	2016
195	NPA	EV-D68	21	2016
196	NPA	EV-D68	20.3	2016
197	NPA	EV-D68	32.2	2016
198	NPA	EV-D68	25.4	2016
199	NP	EV-D68	28.1	2016
200	NPA	EV-D68	23.6	2016
201	NA	EV-D68	18.7	2016
202	NP	EV-D68	22.7	2016
203	NP	EV-D68	30.6	2016
204	NP	EV-D68	25.2	2016
205	NPA	EV-D68	28.9	2016
206	NP	EV-D68	27.8	2016
207	NP	EV-D68	27.8	2016
208	NP	EV-D68	26.7	2016
209	NA	EV-D68	33	2016
210	NPA	EV-D68	26.9	2016
211	NP	EV-D68	30.8	2016
212	NW	EV-D68	22.2	2016
213	NP	EV-D68	27.8	2016

214	NP	EV-D68	31.4	2016
215	NA	EV-D68	18.6	2016
216	NPA	EV-D68	27.4	2016
217	NP	EV-D68	30.4	2016
218	NP	EV-D68	29.6	2016
219	NP	EV-D68	32.7	2016
220	NP	EV-D68	19.7	2016
221	NP	EV-D68	29	2016
222	NP	EV-D68	32	2016
223	NP	EV-D68	28.2	2016
224	NP	EV-D68	26	2016
225	NP	EV-D68	34.2	2016
226	NP	EV-D68	27.3	2016
227	NP	EV-D68	35	2016
228	NP	EV-D68	24.1	2016
229	NP	EV-D68	30.8	2016
230	NP	EV-D68	30.1	2016
231	NP	EV-D68	29.6	2016
232	NP	EV-D68	23.6	2016
233	NP	EV-D68	28.6	2016
234	NP	EV-D68	22.5	2016
235	NP	EV-D68	21.7	2016
236	NP	EV-D68	24.4	2016
237	NP	EV-D68	31.4	2016
238	NP	EV-D68	30.8	2016
239	NP	EV-D68	37.6	2016
240	NP	EV-D68	29.4	2016
241	NP	EV-D68	29.2	2016
242	NP	EV-D68	28.8	2016
243	NP	EV-D68	29.6	2016
244	NP	EV-D68	29.9	2016
245	NP	EV-D68	31.6	2016
246	NP	EV-D68	36.5	2016
247	NP	EV-D68	31.3	2016
248	NP	EV-D68	37.8	2016
249	NP	EV-D68	28.6	2016
250	NP	EV-D68	26.7	2016
251	NP	EV-D68	26.6	2016
252	NP	EV-D68	23.8	2016
253	NP	EV-D68	22	2017
254	NP	EV-D68	26	2017
255	NP	EV-D68	26.8	2017
256	NP	EV-D68	25.6	2017
257	NP	EV-D68	22.8	2017
258	NP	EV-D68	22.4	2017
259	NP	EV-D68	26.6	2017
260	NP	EV-D68	27	2017
261	NP	EV-D68	28.5	2017
262	NP	EV-D68	23	2017
263	NP	EV-D68	28.9	2017
264	NP	EV-D68	32.6	2018
265	NP	EV-D68	32	2018
266	NP	EV-D68	23.5	2018
267	NP	EV-D68	26.9	2018
268	NP	EV-D68	21	2012

269	NP	RV-A45	Negative	2018
270	NP	Negative	Negative	2018
271	NP	Negative	Negative	2018
272	NP	Negative	Negative	2018
273	NPA	Negative	Negative	2018
274	NP	Negative	Negative	2018
275	NP	EV-D68	36.02	2018
276	NP	EV-D68	35.55	2018
277	NP	EV-D68	28.78	2018
278	NP	EV-D68	31	2018
279	NP	Negative	Negative	2018
280	NP	Negative	Negative	2018
281	NP	Negative	Negative	2018
282	NP	EV-D68	25.03	2018
283	NS	Negative	Negative	2018
284	NP	Negative	Negative	2018
285	NP	Negative	Negative	2018
286	NP	Negative	Negative	2018
287	NP	Negative	Negative	2018
288	NP	Negative	Negative	2018
289	TS	EV-A71	Negative	2018
290	TS	EV-A71	Negative	2018
291	TS	EV-A71	Negative	2018
292	TS	Negative	Negative	2018
293	NP	Negative	Negative	2018
294	NP	Negative	Negative	2018
295	NP	Negative	Negative	2018
296	NP	Negative	Negative	2018
297	NP	Negative	Negative	2018
298	NP	Negative	Negative	2018
299	NP	Negative	Negative	2018
300	NP	Negative	Negative	2018
301	NP	Negative	Negative	2018
302	NP	Negative	Negative	2018
303	NP	Negative	Negative	2018
304	NP	RV-A81	Negative	2018
305	NPA	EV-D68	26.54	2018
306	SP	EV-D68	31.95	2018
307	NP	Negative	Negative	2018
308	NP	EV-D68	24.25	2018
309	NP	Negative	Negative	2018
310	NP	EV-D68	23.24	2018
311	NP	Negative	Negative	2018
312	TS	EV-D68	29.38	2018
313	NP	EV-D68	29.86	2018
314	NP	Negative	Negative	2018
315	NP	EV-D68	28.9	2018
316	NP	Negative	Negative	2018
317	NPA	EV-D68	35.11	2018
318	NPA	Negative	Negative	2018
319	NPA	Negative	Negative	2018
320	TA	Negative	Negative	2018
321	NP	RV-B4	Negative	2018
322	NP	Negative	Negative	2018
323	NP	Negative	Negative	2018

324	NP	Negative	Negative	2018
325	NP	Negative	Negative	2018
326	NP	Negative	Negative	2018
327	NP	Negative	Negative	2018
328	NP	RV-A56	Negative	2018
329	NP	EV-D68	29.62	2018
330	NP	RV-B104	Negative	2018
331	NP	EV-D68	18.27	2018
332	NP	RV-A38	Negative	2018
333	TS	EV-A71	Negative	2018
334	TS	CV-A5	Negative	2018
335	NP	Negative	Negative	2018
336	NP	RV-A40	Negative	2018
337	TS	EV-A71	Negative	2018
338	TS	EV-A71	Negative	2018
339	NP	EV-D68	17.6	2018
340	NP	EV-D68	20.9	2018
341	NP	EV-D68	18.6	2018
342	NP	EV-D68	18.9	2018
343	NP	EV-D68	17.9	2018
344	NP	EV-D68	19.5	2018
345	NP	EV-D68	19.7	2018
346	NP	EV-D68	32.1	2018
347	NP	EV-D68	22.6	2018
348	NP	EV-D68	15.6	2018
349	NP	Negative	Negative	2018
350	NP	EV-D68	27.28	2018
351	NP	RV-A90	Negative	2018
352	NP	EV-D68	31	2018
353	NP	Negative	Negative	2018
354	NP	Negative	Negative	2018
355	NP	EV-D68	36.61	2018
356	NP	EV-D68	30.82	2018
357	NP	Negative	Negative	2018
358	NP	RV-A54	Negative	2018
359	NP	Negative	Negative	2018
360	NP	RV-A101	Negative	2018
361	NP	RV-B4	Negative	2018
362	NP	Negative	Negative	2018
363	NP	Negative	Negative	2018
364	NP	EV-D68	25.14	2018
365	NP	Negative	Negative	2018
366	NW	EV-D68	31.84	2018
367	NW	EV-D68	30.57	2018
368	TS	Negative	Negative	2018
369	NP	Negative	Negative	2018
370	NP	EV-D68	30.96	2018
371	NP	Negative	Negative	2018
372	TS	Negative	Negative	2018
373	NP	Negative	Negative	2018
374	NP	Negative	Negative	2018
375	NP	EV-D68	24.04	2018
376	NP	EV-D68	31.94	2018
377	NP	Negative	Negative	2018
378	NP	Negative	Negative	2018

379	NP	Negative	Negative	2018
380	NP	Negative	Negative	2018
381	NP	Negative	Negative	2018
382	TS	Negative	Negative	2018
383	NP	Negative	Negative	2018
384	NP	Negative	Negative	2018
385	NP	Negative	Negative	2018
386	NP	Negative	Negative	2018
387	NP	EV-D68	32.11	2018
388	NP	Negative	Negative	2018
389	NP	Negative	Negative	2018
390	NP	Negative	Negative	2018
391	NPA	EV-D68	29.18	2018
392	NPA	EV-D68	36.23	2018
393	NP	Negative	Negative	2018
394	NP	Negative	Negative	2018
395	NP	Negative	Negative	2018
396	NP	Negative	Negative	2018
397	NP	EV-D68	24.63	2018
398	NP	Negative	Negative	2018
399	TS	EV-D68	35.46	2018
400	NP	Negative	Negative	2018
401	NP	Negative	Negative	2018
402	NS	RV-B4	Negative	2018
403	NP	Negative	Negative	2018
404	NP	Negative	Negative	2018
405	TS	Negative	Negative	2018
406	NP	Negative	Negative	2018
407	NP	EV-D68	26.46	2018
408	NP	Negative	Negative	2018
409	NP	Negative	Negative	2018
410	NP	Negative	Negative	2018
411	NP	Negative	Negative	2018
412	NP	Negative	Negative	2018
413	NP	Negative	Negative	2018
414	NP	EV-D68	24.95	2018
415	NP	Negative	Negative	2018
416	NP	CV-A6	Negative	2018
417	NP	CV-A6	Negative	2018
418	NP	Negative	Negative	2018
419	NP	Negative	Negative	2018
420	NP	CV-A6	Negative	2018
421	NP	Negative	Negative	2018
422	NP	Negative	Negative	2018
423	NP	Negative	Negative	2018
424	NP	RV-A85	Negative	2018
425	NP	Negative	Negative	2018
426	NP	Negative	Negative	2018
427	TS	RV-A104	Negative	2018
428	NP	Negative	Negative	2018
429	NP	Negative	Negative	2018
430	NP	Negative	Negative	2018
431	NP	Negative	Negative	2018
432	NP	Negative	Negative	2018
433	NP	Negative	Negative	2018

434	NP	Negative	Negative	2018
435	NP	Negative	Negative	2018
436	NW	EV-D68	32.7	2018
437	NP	Negative	Negative	2018
438	NP	Negative	Negative	2018
439	NP	Negative	Negative	2018
440	NP	Negative	Negative	2018
441	NS	Negative	Negative	2018
442	NP	Negative	Negative	2018
443	NP	Negative	Negative	2018
444	NW	Negative	Negative	2018
445	NW	Negative	Negative	2018
446	NPA	EV-D68	29.7	2018
447	NP	Negative	Negative	2018
448	NP	Negative	Negative	2018
449	NP	Negative	Negative	2018
450	NP	Negative	Negative	2018
451	NP	Negative	Negative	2018
452	NP	Negative	Negative	2018
453	NP	Negative	Negative	2018
454	TS	Negative	Negative	2018
455	NP	Negative	Negative	2018
456	NW	EV-D68	25.1	2018
457	NP	RV-A101	Negative	2018
458	NP	RV-A22	Negative	2018
459	NP	Negative	Negative	2018
460	NP	EV-D68	28.9	2018
461	NP	EV-D68	33.6	2018
462	NP	RV-A12	Negative	2018
463	NP	Negative	Negative	2018
464	NW	EV-D68	29.3	2018
465	NS	EV-D68	32.9	2018
466	TS	Negative	Negative	2018
467	NP	Negative	Negative	2018
468	NP	Negative	Negative	2018
469	NP	Negative	Negative	2018
470	NP	Negative	Negative	2018
471	NP	Negative	Negative	2018
472	NP	Negative	Negative	2018
473	NP	EV-D68	32.6	2018
474	NP	Negative	Negative	2018
475	NP	Negative	Negative	2018
476	SP	EV-D68	30	2018
477	NP	EV-D68	27.5	2018
478	NP	Negative	Negative	2018
479	NP	Negative	Negative	2018
480	NP	Negative	Negative	2018
481	TS	E-11	Negative	2018
482	TS	EV-A71	Negative	2018
483	NP	Negative	Negative	2018
484	NP	Negative	Negative	2018
485	NP	Negative	Negative	2018
486	NW	EV-D68	33.3	2018
487	NW	Negative	Negative	2018
488	NP	Negative	Negative	2018

489	NP	Negative	Negative	2018
490	NP	Negative	Negative	2018
491	NAS	Negative	Negative	2018
492	NP	Negative	Negative	2018
493	NP	Negative	Negative	2018
494	NAS	Negative	Negative	2018
495	NP	CV-B2	Negative	2018
496	NP	Negative	Negative	2018
497	NP	Negative	Negative	2018
498	NP	RV-A8	Negative	2018
499	TS	EV-D68	30.9	2018
500	NP	Negative	Negative	2018
501	NP	Negative	Negative	2018
502	NP	Negative	Negative	2018
503	NP	Negative	Negative	2018
504	NP	Negative	Negative	2018
505	NP	Negative	Negative	2018
506	NP	Negative	Negative	2018
507	NP	RV-A16	Negative	2018
508	NP	Negative	Negative	2018
509	NP	Negative	Negative	2018
510	NW	Negative	Negative	2018
511	NP	Negative	Negative	2018
512	OP	Negative	Negative	2018
513	NP	Negative	Negative	2018
514	NP	Negative	Negative	2018
515	NP	Negative	Negative	2018
516	NP	Negative	Negative	2018
517	NAS	Negative	Negative	2018
518	NP	CV-A6	Negative	2018
519	NP	Negative	Negative	2018
520	NP	Negative	Negative	2019
521	NP	Negative	Negative	2019
522	NP	Negative	Negative	2019
523	NP	Negative	Negative	2019
524	NP	Negative	Negative	2019
525	NP	Negative	Negative	2019
526	NW	Negative	Negative	2019
527	NP	Negative	Negative	2019
528	NP	Negative	Negative	2019
529	NAS	Negative	Negative	2019
530	NP	Negative	Negative	2019
531	NP	Negative	Negative	2019
532	NP	Negative	Negative	2019
533	NP	Negative	Negative	2019
534	NP	Negative	Negative	2019
535	NP	Negative	Negative	2019
536	NP	Negative	Negative	2019
537	NP	Negative	Negative	2019
538	NP	Negative	Negative	2019
539	NP	Negative	Negative	2019
540	NP	Negative	Negative	2019
541	NP	Negative	Negative	2019
542	NP	Negative	Negative	2019
543	NP	Negative	Negative	2019

544	NP	Negative	Negative	2019
545	NP	Negative	Negative	2019
546	NP	Negative	Negative	2019
547	NP	Negative	Negative	2019
548	NPA	Negative	Negative	2019
549	NP	Negative	Negative	2019
550	NP	Negative	Negative	2019
551	NP	Negative	Negative	2019
552	NP	Negative	Negative	2019
553	BW	Negative	Negative	2019
554	NP	Negative	Negative	2019
555	NP	Negative	Negative	2019
556	NP	Negative	Negative	2019
557	NP	Negative	Negative	2019
558	NP	RV-A12	Negative	2019
559	BW	RV-A12	Negative	2019
560	BW	Negative	Negative	2019
561	NP	Negative	Negative	2019
562	NP	Negative	Negative	2019
563	NP	Negative	Negative	2019
564	NP	Negative	Negative	2019
565	NP	Negative	Negative	2019
566	NP	Negative	Negative	2019
567	TA	Negative	Negative	2019
568	NP	RV-A34	Negative	2019
569	NP	Negative	Negative	2019
570	NP	Negative	Negative	2019
571	NP	Negative	Negative	2019
572	NP	Negative	Negative	2019
573	NP	Negative	Negative	2019
574	NW	Negative	Negative	2019
575	NP	EV-D68	EV-D68(+)	2018
576	NPA	EV-D68	EV-D68(+)	2018
577	NP	EV-D68	EV-D68(+)	2018
578	NP	EV-D68	EV-D68(+)	2018
579	TS	EV-D68	EV-D68(+)	2018
580	NP	EV-D68	EV-D68(+)	2018
581	TW	EV-D68	EV-D68(+)	2018
582	NP	EV-D68	EV-D68(+)	2018
583	NP	EV-D68	EV-D68(+)	2018
584	NP	EV-D68	EV-D68(+)	2018
585	NP	EV-D68	EV-D68(+)	2018
586	NP	EV-D68	EV-D68(+)	2018
587	NP	EV-D68	EV-D68(+)	2018
588	NP	EV-D68	EV-D68(+)	2018
589	NP	EV-D68	EV-D68(+)	2018
590	NP	EV-D68	EV-D68(+)	2018
591	NP	EV-D68	EV-D68(+)	2018
592	NP	EV-D68	EV-D68(+)	2018
593	NP	EV-D68	EV-D68(+)	2018
594	NP	EV-D68	EV-D68(+)	2018
595	NP	EV-D68	EV-D68(+)	2018
596	NW	EV-D68	EV-D68(+)	2018
597	NW	EV-D68	EV-D68(+)	2018
598	NP	EV-D68	EV-D68(+)	2018

599	NP	EV-D68	EV-D68(+)	2018
600	NP	EV-D68	EV-D68(+)	2018
601	NPA	EV-D68	EV-D68(+)	2018
602	NPA	EV-D68	EV-D68(+)	2018
603	NP	EV-D68	EV-D68(+)	2018
604	NP	EV-D68	EV-D68(+)	2018
605	NPA	EV-D68	EV-D68(+)	2018
606	NW	EV-D68	EV-D68(+)	2018
607	NP	EV-D68	EV-D68(+)	2018
608	NP	EV-D68	EV-D68(+)	2018
609	NS	EV-D68	EV-D68(+)	2018
610	NP	EV-D68	EV-D68(+)	2018
611	SP	EV-D68	EV-D68(+)	2018
612	NP	EV-D68	EV-D68(+)	2018
613	TS	EV-D68	EV-D68(+)	2018
614	NP	EV-D68	EV-D68(+)	2019
615	NP	EV-D68	EV-D68(+)	2022
616	NP	EV-D68	EV-D68(+)	2022
617	NP	EV-D68	EV-D68(+)	2022
618	NP	EV-D68	EV-D68(+)	2022
619	NP	EV-D68	EV-D68(+)	2022
620	NP	EV-D68	EV-D68(+)	2022
621	NP	EV-D68	EV-D68(+)	2022
622	NP	EV-D68	EV-D68(+)	2022
623	NP	EV-D68	EV-D68(+)	2022
624	NP	EV-D68	EV-D68(+)	2022
625	NP	EV-D68	EV-D68(+)	2022
